# The Ribosome Can Prevent Aggregation of Partially Folded Protein Intermediates: Studies Using the *Escherichia coli* Ribosome

**DOI:** 10.1371/journal.pone.0096425

**Published:** 2014-05-07

**Authors:** Bani Kumar Pathak, Surojit Mondal, Amar Nath Ghosh, Chandana Barat

**Affiliations:** 1 Department of Biotechnology, St. Xavier’s College, Kolkata, West Bengal, India; 2 National Institute of Cholera and Enteric Diseases P-33, Scheme XM, Beleghata, India; University of Arkansas for Medical Sciences, United States of America

## Abstract

**Background:**

Molecular chaperones that support de novo folding of proteins under non stress condition are classified as chaperone ‘foldases’ that are distinct from chaperone’ holdases’ that provide high affinity binding platform for unfolded proteins and prevent their aggregation specifically under stress conditions. Ribosome, the cellular protein synthesis machine can act as a foldase chaperone that can bind unfolded proteins and release them in folding competent state. The peptidyl transferase center (PTC) located in the domain V of the 23S rRNA of *Escherichia coli* ribosome (bDV RNA) is the chaperoning center of the ribosome. It has been proposed that via specific interactions between the RNA and refolding proteins, the chaperone provides information for the correct folding of unfolded polypeptide chains.

**Results:**

We demonstrate using *Escherichia coli* ribosome and variants of its domain V RNA that the ribosome can bind to partially folded intermediates of bovine carbonic anhydrase II (BCAII) and lysozyme and suppress aggregation during their refolding. Using mutants of domain V RNA we demonstrate that the time for which the chaperone retains the bound protein is an important factor in determining its ability to suppress aggregation and/or support reactivation of protein.

**Conclusion:**

The ribosome can behave like a ‘holdase’ chaperone and has the ability to bind and hold back partially folded intermediate states of proteins from participating in the aggregation process. Since the ribosome is an essential organelle that is present in large numbers in all living cells, this ability of the ribosome provides an energetically inexpensive way to suppress cellular aggregation. Further, this ability of the ribosome might also be crucial in the context that the ribosome is one of the first chaperones to be encountered by a large nascent polypeptide chains that have a tendency to form partially folded intermediates immediately following their synthesis.

## Introduction

Protein folding in biological cells is not yet well understood. Following ribosome mediated synthesis of the proteins the polypeptide chains are released into a highly crowded cellular environment where they require the assistance of a number of molecular chaperones to either fold or be rescued from misfolding and aggregation. The ribosome associated molecular chaperones like the complex of Hsp70 and J-type chaperones in the yeast *Saccharomyces cerevisiae* and Trigger factor in *Escherichia coli* ensure that the nascent polypeptide chain is kept in a folding competent state until the whole sequence information is available [1]. The ribosome, the polypeptide synthesis machinery itself, has chaperoning abilities and is capable of assisting in folding of proteins. The chaperoning activity originates in the domain V of the 23S rRNA (bDV RNA) (Figure S1A) of *E. coli* ribosome [2]. Since the large polypeptide chains that constitute a significant component of the cell’s proteome fold via formation of intermediate [3], these proteins are likely to collapse into their partially folded forms in the crowded cellular environment immediately following their synthesis. The first chaperone to be encountered by these partially folded protein intermediates is likely to be the ribosome since it is itself the site for polypeptide synthesis.

All earlier studies on ribosome assisted folding were performed on completely unfolded state of proteins [2]. The purpose of the present study was to investigate the ability of *E.coli* ribosome and the domain V of its 23S rRNA to interact with a partially folded intermediate state of proteins and influence their aggregation and reactivation under refolding conditions. Our studies were performed on the proteins a) bovine carbonic anhydrase II (BCAII) which under mild denaturing conditions assumes an equilibrium molten globule state [4] and b) chicken egg white lysozyme that forms a hydrophobically collapsed state at the onset of its folding process that have properties characteristic of equilibrium molten globule [5]–[9].

The protein folding ability of ribosome appears to be a universal one and have been demonstrated with ribosome isolated from wide range of sources including the eubacteria, archaebacteria, eukaryotes (rat liver, wheat germ, yeast), rabbit reticulocyte, bovine mitochondria and mitochondria of the parasite *Leishmenia donovani* [10]–[16]. The large ribosomal subunit is attributed with the chaperoning ability and like the peptidyl transferase ability; the chaperoning activity of the ribosome also originates in the ribosomal RNA of the ribonucleoprotein complex. The domain V of 23S rRNA of bacterial large ribosomal subunit that houses the peptidyl transferase function of ribosome is also its chaperoning center [2]. The RNA corresponding to domain V of 23S rRNA of *E. coli* ribosome synthesized by in vitro transcription also possess chaperoning ability. Studies on the mechanisms of domain V chaperoning activity showed that it is a two step process involving its two sub-domains RNA1 and RNA2 [17]. The initial binding of the unfolded proteins take place with the RNA1 sub-domain that is the central region of PTC and although the substrate proteins possess no apparent RNA binding domain, they interact with the RNA1 region of domain V RNA via specific interactions [18]. The RNA2 region of this domain is responsible for the releasing the bound protein which subsequently folds into its native structure [17]. The domain V of large subunit rRNA of bovine mitochondrial ribosome (mDV RNA) has a truncated RNA2 region (Figure S1C) and therefore shows a delay in releasing the bound protein [15]. Unlike other cellular foldases neither the binding nor the release steps are associated with ATP hydrolysis.

The ability of the chaperones to interact with partially folded intermediates of proteins is well documented. The chaperonin GroEL primarily recognizes contiguous sequence elements or hydrophobic surfaces, such as those typically exposed in the molten globule intermediates that form in the early stages of folding due to the partial collapse of the hydrophobic residues [19], [20]. The chaperone alpha-crystallin is capable of interacting with aggregation prone refolding intermediate of lysozyme and can also form stable complexes with the molten globule state of alpha-lactalbumin and carbonic anhydrase [21]–[23]. Here we have investigated the ribosome, its domain V RNA and variants of domain V RNA in terms of their ability to influence reactivation or aggregation during refolding of a) molten globule state of BCAII and b) reduced-denatured lysozyme using enzymatic assays, turbidity measurements, electron microscopy, filter binding studies and gel filtration chromatography.

Our studies show that the ribosome, more specifically domain V of 23S rRNA can interact with a range of different folded states of the BCAII and influence their reactivation. We also demonstrate that these chaperones can interact with and prevent aggregation of BCAII and lysozyme molten globule. Using variants of domain V RNA and its mutants, we demonstrate that the time for which the chaperone retains the bound protein is an important factor in determining its ability to suppress aggregation and that the reactivation of protein and suppression of aggregation might represent two distinct properties of the chaperone.

## Materials and Methods

### Materials

Bovine carbonic anhydrase II (BCAII), hen egg white lysozyme, Ribonuclease A, *Micrococcus lysodeikticus,* cystine hydrochloride, fluorescein isothiocyanate (FITC), Guanidine hydrochloride (GuHCl), dithiothreitol (DTT), GTP, ATP and antibiotics that specifically bind to domainV of 23S rRNA (blasticidin and chloramphenicol) were purchased from Sigma. Nitrocellulose filter was purchased from Millipore, p-nitrophenylacetate (p-NPA) from SRL biochemical and reagents for molecular biology like T7 RNA Polymerase and RNase free DNase I were purchased from Fermentas, Ni^+2^-NTA was purchased from Qiagen. All other chemicals were local products of analytical grade. All data analysis was performed using OriginPro 8 software.

### Buffers

The following buffers were used : BCAII refolding buffer, 50 mM Tris-HCl (pH 7.5), 10 mM MgCl_2_, 100 mM NaCl; blasticidin binding buffer, 100 mM Tris-HCl (pH 7.2), 10 mM MgCl_2_, 100 mM NH_4_Cl (pH 7.2), 6 mM β-Mercaptoethanol; [24], chloramphenicol binding buffer, 20 mM Tris-HCl (pH 7.5), 10 mM MgCl_2,_ 50 mM NH_4_Cl_,_100 mM KCl; [25], lysozyme refolding buffers (non-redox buffer- 50 mM Tris-HCl, pH 7.5, 100 mM NaCl, 10 mM MgCl_2_, 1 mM DTT; redox buffer- non-redox buffer containing 1 mM cystine hydrochloride).

### Denaturation and Refolding of BCAII

**Figure 1:**
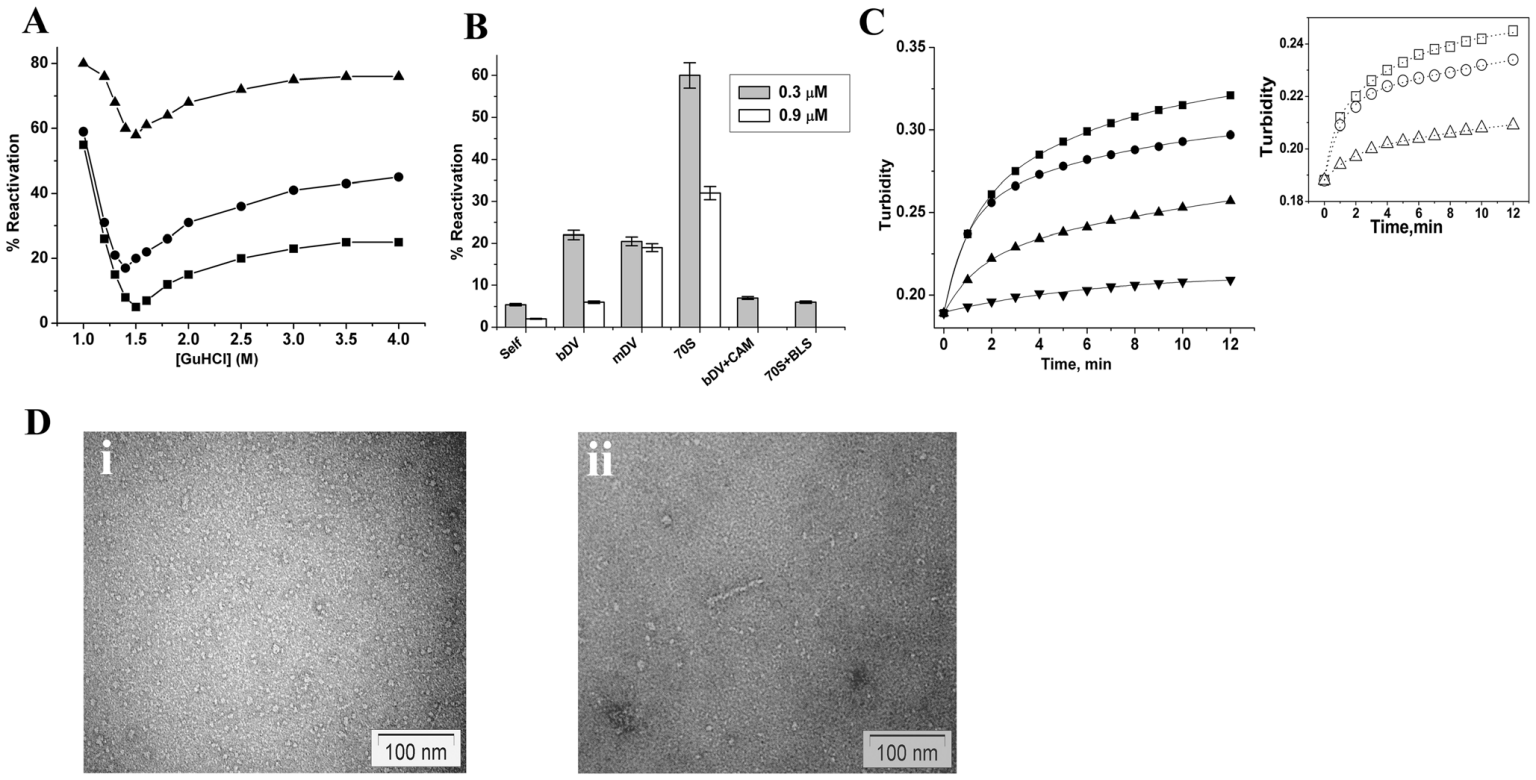
Effect of ribosome and domain V RNA on refolding of BCAII. A) Reactivation yields of various GuHCl denatured (1–4 M) BCAII after 30 min of incubation in the absence of chaperone (-▪-), presence of 70S ribosome (-▴-), and bacterial domain V RNA (-•-). B) Bar graphs showing the reactivation of BCAII-m at lower (0.3 µM; fill bar) or higher (0.9 µM; blank bar) protein concentrations after 60 minute of incubation with or without the chaperones as marked in the figure. Effect of bDV RNA specific antibiotics, chloramphenicol (bDV+CAM) and blasticidin (70S+BLS) on BCAII-m reactivation are also shown. Bar graphs represent the mean reactivation values (± standard deviations) from three independent experiments. C) Time course of change in turbidity at 320 nm of BCA II-m in BCAII refolding mix in absence of chaperone (-▪-), in presence of bDV RNA (-•-), in presence of mDV RNA (-▴-), are shown at 0.9 µM protein concentration. The change in turbidity at 0.3 µM protein concentration (-▾-) is also shown in this figure. The inset shows the time course of change of turbidity at 450 nm in refolding condition in absence of chaperone (-□-),in presence of bDV RNA (-○-) and in presence of ribosome (-Δ-). D) Negative staining transmission electron micrographs of BCAII-m refolding in absence (i) and in presence of mDV RNA (ii). BCAII denatured with 1.5 M GuHCl was diluted 100 folds in presence of mDV RNA. Molar ratio of BCAII to mDV RNA is 1∶1.

Ribosomes were purified from *E. coli* MRE 600 cells [14] and the RNA corresponding domain V of the ribosome were synthesized by run-off transcription and prepared as described earlier [15], [26]. Studies on the effect of these chaperones on refolding of the enzyme BCAII was performed as reported earlier [2]. Briefly, 30 µM of BCAII was denatured with various concentrations of Guanidine hydrochloride (GuHCl) (1 M- 4 M) in presence of 3.5 mM EDTA for 2.5 hour. The denatured protein was diluted 100 times in BCAII refolding buffer (see above) to achieve final protein concentration 0.3 µM, incubated at 29°C for a period of 30 min or 60 min as indicated and recovery of enzymatic activity assayed from the absorbance at 420 nm using p-nitrophenylacetate as substrate. The activity of similar amount of the native protein was assumed as 100% for calculation of reactivation yields. The chaperone and its variants used in this study are: the *E.coli* 70S ribosome, blasticidin bound ribosome, bDV RNA, mutants of bDV RNA, chloramphenicol bound bDV RNA, mDV RNA (Fig. 1), recombinant DnaK and Trigger factor. In all the refolding studies BCAII and the chaperones (ribosome, bDV RNA and its mutants, mDV RNA, DnaK and Trigger factor) are present at equimolar concentration (0.3 µM). Ribosome bound antibiotic complex were prepared by incubating 0.3 µM ribosome with either 2 mM chloramphenicol or 10 µM blasticidin in 297 µl of respective binding buffer (see above) at 37°C for 20 min and then at 20°C for 15 min. Refolding of BCAII in presence of antibiotic bound ribosome complex was performed as described above. Care was taken to ensure that unassisted self and ribosome assisted refolding were also performed under the same salt and buffer conditions. BCAII denatured with 1.5 M GuHCl assumes an equilibrium molten globule state and is referred to as BCAII-m in this paper. The final concentrations of BCAII-m during its refolding were either 0.3 µM or 0.9 µM as is specified in the text or the figure legends.

Aggregation of BCAII-m (0.9 µM) was monitored by turbidity measurement in Hitachi spectrophotometer (U-1900). The effect of bDV RNA and mDV RNA on BCAII-m aggregation was monitored at 320 nm while the effect of ribosome was monitored at 450 nm since the ribosome itself interferes with turbidity measurements at 320 nm. All measurements were repeated three times and the data represents the average of all these experiments.

### Denaturation and Refolding of Lysozyme

Lysozyme (200 µM) was completely reduced and denatured by incubation at room temperature for 3 hours in presence of 6 M GuHCl and 100 mM DTT [27]. Refolding of reduced-denatured lysozyme was initiated at 30°C by 100 fold dilution in 300 µl of non-redox (50 mM Tris-HCl, pH 7.5, 100 mM NaCl, 10 mM MgCl_2_, final DTT concentration 1 mM) or redox (non-redox buffer containing 1 mM cystine hydrochloride) buffer systems to achieve final protein concentration 2 µM. During refolding lysozyme and the chaperones (ribosome, bDV RNA, mDV RNA, DnaK and Trigger factor) were present at equimolar concentrations (2 µM). The effect on the reactivation yields of reduced-denatured lysozyme under redox conditions in presence of a) RNase treated bDV RNA and b) ribosome treated with Proteinase K, extracted using phenol, precipitated with ethanol and treated with RNase were also determined. The refolding mix was incubated at 30°C for a period of 16 hrs. Recovery of enzymatic activity was determined at 30°C by following the lysis of *Micrococcus lysodeikticus* [28]. The decrease in absorbance at 450 nm of a 0.25 mg.mL^−1^ cell suspension was measured in a Hitachi UV-visible spectrophotometer. The activity of similar amount of native lysozyme was assumed as 100% for calculating the percent reactivations obtained in our experiments. Aggregation of lysozyme was monitored by turbidity measurement at 450 nm in a Hitachi spectrophotometer in a final concentration of 2 µM.

### In-vitro Synthesis of Ribosomal RNA

Earlier studies have shown that bDV RNA mediated folding is a two step process involving its two segments (Figure S1) [17], [18]. Initial interaction of unfolded proteins with RNA1 region is followed by RNA2 mediated release of the protein in a folding competent form. The RNA fragments corresponding to this region when synthesized separately retained these properties and could complement each other in the chaperoning function. The RNA1 bound protein could also be released in presence of 3% ethanol [17] **.** The PTC of bovine mitoribosomal large rRNA (mDV RNA), that has major deletions in the RNA2 region shows delayed release and reactivation of the bound protein (60 min with mDV RNA vs 30 min with bDV RNA) [15]. The DNA corresponding to bDV (625 bp) and mDV (450 bp), cloned in plasmid pTZ57R/T were kind gifts from laboratory of Professor C. Dasgupta (Univ. of Calcutta). The DNA corresponding to RNA1 and RNA2 portions of *E.coli* domain V were cloned into the pTZ57R/T vector downstream to the T7 Polymerase promoter. The RNA corresponding to mDV RNA, bDV RNA, mutants of bDV RNA (see below), RNA1 and RNA2 were synthesized by run-off transcription and prepared as described earlier [15], [26].

### Site Directed Mutagenesis

Mutations U2585C (in the RNA1 region) and delG2252 (in the RNA2 region) were introduced into DNA corresponding to bDV RNA, bDV RNA1 and bDV RNA2 were generated using appropriate primers by site directed mutagenesis kit (Stratagene). Mutations were confirmed by sequencing.

### Cloning and Purification of DnaK and Trigger Factor

The DNA corresponding to chaperones DnaK and Trigger factor were PCR amplified using *E. coli* genomic DNA extracted from *E. coli* (MG 1655) cells as template, Pfu DNA Polymerase and appropriate primers. The PCR amplified products were cloned into the pET-28a (+) (NOVAGEN) expression vector. The gene now contains a T7 promoter upstream of a ribosome binding site with the “epsilon sequence” originating from bacteriophage T7 promoter followed by a Shine–Dalgarno sequence. The *E.coli* BL21-DE3 cells transformed with the recombinant plasmids were grown and induced with 0.5 mM IPTG for 4 hours, the cells were centrifuged at 4°C at 8000 rpm for 15 minutes. Cell pellet was washed by wash buffer containing 50 mM Tris-HCl (pH 7.5), 200 mM KCl, 1 mM β-mercaptoethanol and disrupted by sonication. Cell debris was pelleted by centrifugation for 45 minutes at 12,000 r.p.m. The supernatant was loaded on a Ni^+2^–NTA affinity flow column and eluted with a linear gradient of imidazole (30–300 mM) in wash buffer. Eluted fractions (corresponding to different imidazole wash) were subjected to SDS-PAGE with appropriate protein ladder. Selected fractions corresponding to DnaK or Trigger factor were pooled separately, dialyzed and protein concentration was estimated by measuring the absorbance at 280 nm.

### Electron Microscopy

BCAII-m or reduced-denatured lysozyme was diluted in refolding buffer in presence of the mDV RNA as stated above. Imaging of aggregation in the refolding samples was done by using a transmission electron microscope with an acceleration voltage of 120 kV. Aliquots (5 µl) of refolding solution containing the protein with or without the domain V RNA chaperone were placed on the copper grid coated with carbon film (300 meshes) and one drop of 2% uranyl acetate was placed on the grid. The excess water was removed carefully with filter paper and the grid was left to dry in air.

### Binding and Release of BCAII and Lysozyme with Ribosome and Domain V RNA and its Mutants

#### Filter binding studies

Filter binding studies were performed as described in earlier studies [26]. The wild-type and mutant RNA were labeled with radio-isotope [α-^32^P] UTP during in vitro transcription. Reduced-denatured lysozyme or BCAII-m was incubated with equimolar radiolabeled RNA at 4°C for different time intervals. After incubation the samples were cross-linked by UV irradiation at 254 nm for 90 seconds (GS GeneLinker, Bio-Rad) and filtered through pre-soaked nitrocellulose filter paper (Millipore) with pore size of 0.22 µM. The filter papers were dried and ^32^P counts were taken in a liquid scintillation counter (Perkin Elmer). The RNA bound to denatured protein was retained on the filter while the free RNA passed through it. The percentage of radioactivity retained on the filter paper was calculated and plotted against incubated time. Comparing the radioactive count incorporated in the total RNA to that on the filter, the percentage of radioactivity retained was calculated.

#### Size**-**exclusion chromatography

BCAII and lysozyme were fluorescent labeled with FITC. BCAII-m or reduced-denatured lysozyme was diluted in refolding buffer in presence of the ribosome. Aliquots were withdrawn at 30 seonds after initiation of refolding, UV- cross - linked and analyzed by SEC using a Sephacryl S-300 column (length×diameter = 8 inch×0.6 inch) with refolding buffer as mobile phase (flow rate of 0.250 ml/min). Proteins in the eluted fractions were detected by FITC fluorescence at an excitation wavelength of 494 nm and an emission wavelength of 518 nm and a band pass of 5 nm using a Hitachi 2700 fluorescence detector. Ribosome in the eluted fractions was detected by absorbance at 260 nm in a spectrophotometer. The elution profiles of the protein and the ribosome were plotted together to compare between the ribosome bound and unbound proteins.

## Results

### Effect of Ribosome and Domain V RNA on Refolding of BCAII Molten Globule

#### Reactivation of BCAII-m

Earlier studies have demonstrated the ability of the ribosome to assist in folding of BCAII from its completely unfolded state. To assess the ability of the chaperone to fold the protein from its partially denatured states, the following experiments were performed.

BCAII, denatured in presence of GuHCl was refolded upon rapid dilution of the denaturant in presence of equimolar concentration of the chaperones 70S ribosome, bDV RNA or mDV RNA. The reactivation yields after 30 minutes of refolding were compared to that attained in absence of the chaperones. The final BCAII concentrations during refolding were 0.3 µM or 0.9 µM. As shown in Fig. 1A, the ribosome and bDV RNA is not only capable of binding the completely unfolded form of BCAII as reported earlier, but were also capable of interacting with a range of partially unfolded forms of the protein and increasing reactivation yields during their refolding. The reactivation yields when plotted against the concentration of denaturant have the appearance of a trough as reported earlier [29]. This appearance is also observed in presence of the chaperones. Minimum reactivation is achieved with BCAII denatured in presence of 1.5 M GuHCl at which the protein assumes a partially folded equilibrium “molten globule” form (BCAII-m) [4] **,** [29].

All subsequent studies on the chaperoning ability of ribosome, bDV RNA or mDV RNA were performed with BCAII-m. As shown in Fig. 1B, the reactivation yields obtained after 60 min of refolding either in presence or absence of the chaperones were higher at lower protein concentration. For example, the ability of the ribosome to assist in BCAII-m refolding is reduced from 60% to 32% at higher protein concentration. Although at both BCAII-m concentrations, the ribosome was more effective than its domain V RNA in increasing reactivation yield, complete inhibition of the chaperoning action of ribosome and bDV RNA is observed upon binding to domain V specific antibiotics blasticidin and chloramphenicol respectively. This establishes that the chaperoning action observed here originates in the domain V of 23S rRNA of the ribosome. The Fig. 1B also shows that while at lower protein concentration both bDV and mDV RNA are able to achieve comparable reactivation yields, at a concentration of 0.9 µM, mDV RNA is significantly more effective in assisting refolding than bDV RNA. Interaction between early refolding species of BCAII-m and the bDV RNA chaperone is important since a rapid decline in chaperoning ability is observed upon an increase in delay of chaperone addition (Figure S2).

#### Turbidity measurements

Aggregation of BCAII-m under refolding conditions was followed by turbidity measurements at 320 nm. As shown in Fig. 1C aggregation proceeds rapidly at 0.9 µM protein concentration. The mDV RNA is more effective in suppressing aggregation than bDV RNA, which possibly explains the better refolding yield in presence of mDV RNA, as stated above. Due to interference of the ribosome in turbidity measurements at 320 nm, studies with ribosome were performed at 450 nm. A comparison of the increase in turbidity in presence of bDV RNA and the ribosome is shown in the inset of Fig. 1C. The ribosome is more effective in suppressing aggregation compared to its domain V RNA.

#### Electron microscopy

The suppression of aggregation of BCAII-m observed in presence of mDV RNA was confirmed by electron microscopy. BCAII-m (0.9 µM) was incubated in presence of equimolar concentration of mDV RNA under refolding conditions as stated above. A large number of small BCAII aggregates were observed in absence of the chaperones (Fig. 1D.i). In presence of mDV RNA a reduction in aggregation is observed that is in agreement with the turbidity measurement studies (Fig. 1D.ii).

#### Effect of ribosome associated chaperones on refolding of BCAII

The ribosome is viewed as a ‘platform’ for nascent polypeptide folding [30] **.** Multiple ribosome associated chaperones like the Trigger factor and DnaK primarily ensure improper association of polypeptides during or immediately following their synthesis. A comparison of the ability of these chaperones to increase reactivation or suppress aggregation during refolding of BCAII-m to that of the ribosome is shown in Figure S3A and Figure S3B. These studies were performed in absence of any added co-chaperones or co-factors and the chaperones were present at equimolar ratio with the refolding protein. Addition of DnaK and Trigger factor leads to no further improvement in reactivation yield of BCAII-m over that attained with 70S ribosome.As shown in Figure S3B the ribosome was the more effective compared to both DnaK and Trigger factor in suppressing BCAII aggregation.

### Effect of Ribosome, Domain V on Refolding of Reduced and Denatured Lysozyme

#### Reactivation of reduced and denatured lysozyme

Lysozyme, that has been reduced and denatured, folds via formation of a transient molten globule intermediate [27]. To assess the ability of the 70S ribosome, bDV RNA and mDV RNA to interact with and influence refolding of lysozyme the following experiments were performed.

**Figure 2:**
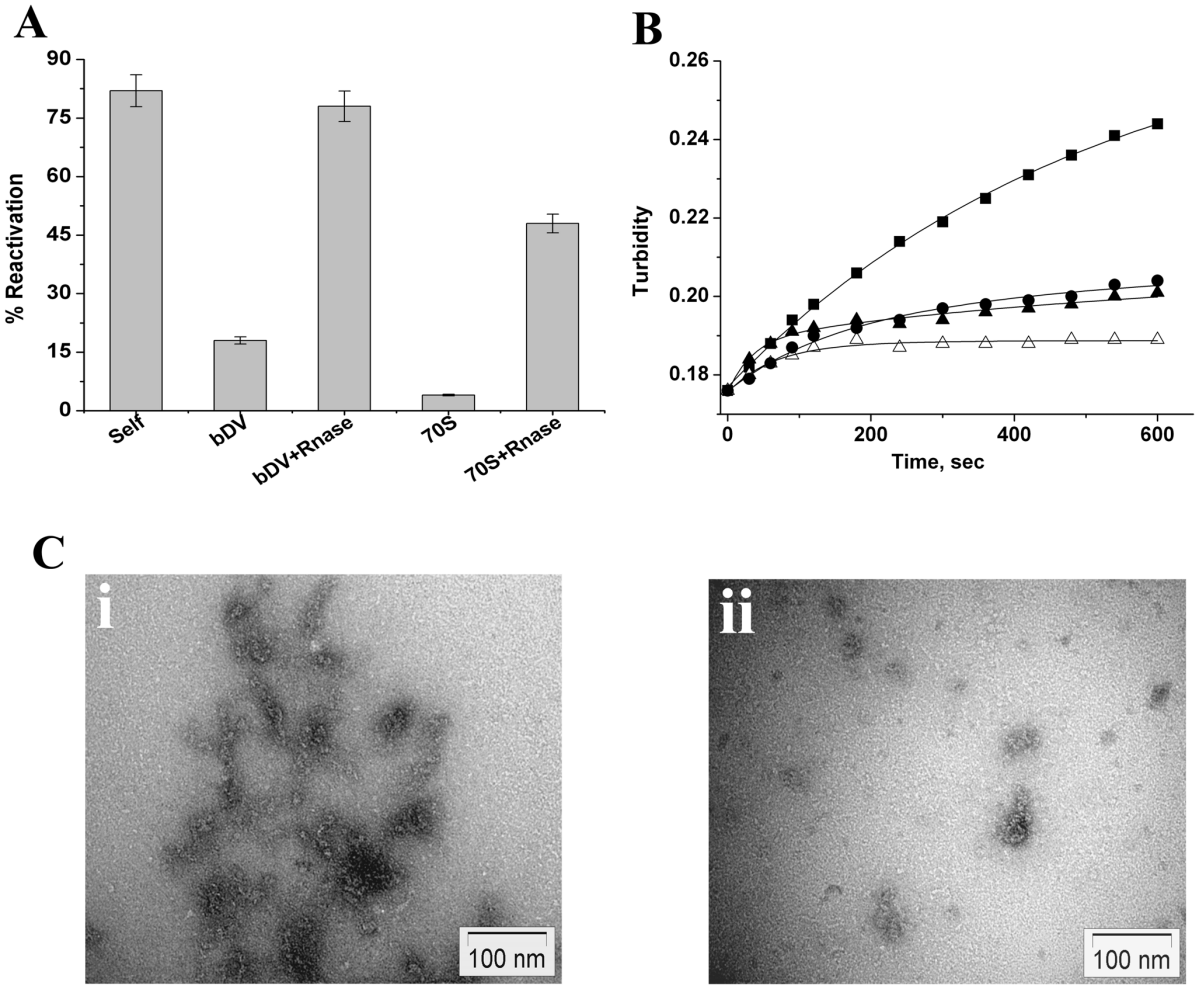
Effect of ribosome and domain V RNA on refolding of lysozyme. A) Reduced-denatured lysozyme (2 µM) was refolded for 16 h in redox buffer (Material and methods). Bar diagram shows the reactivation yields in absence of the chaperone (Self) and in presence of bDV RNA, bDV RNA+Rnase. Reactivation yields in presence of 70S ribosome (70S) and Proteinase K treated and phenol extracted ribosome that was digested with RNase (70S ribosome+Rnase) are also shown. B) Time course of change in turbidity at 450 nm of reduced-denatured lysozyme (2 µM ) upon dilution of denaturant into the non-redox buffer (Material and methods), in absence of chaperone (-▪-), in presence of bDV RNA (-•-), in presence of mDV RNA (-▴-) and 70S ribosome (-Δ-). C) Negative staining transmission electron micrographs of reduced-denatured lysozyme under refolding condition in absence (i) and in presence of bDV RNA (ii). Molar ratio of BCAII to bDV RNA is 1∶1.

Lysozyme was reduced and denatured in presence of 100 mM DTT and 6 M GuHCl and refolded by 100 fold dilution in presence of ribosome and its domain V RNA. The protein: chaperone was present in 1∶1 stoichiometric ratio and the final DTT concentration is 1 mM (non-redox buffer). Spontaneous refolding of reduced and denatured lysozyme under these condition shows only marginal (2% reactivation yield) that remain unaffected in presence of bDV RNA, mDV RNA or ribosome (data not shown). The bar diagram in Fig. 2A shows a comparison of lysozyme reactivation yield in the redox buffer system (in presence of 1 mM cystine hydrochloride in refolding buffer), 16 hours after initiation of refolding. The self reactivation of lysozyme (82%) is suppressed in presence of the *E. coli* ribosome, its bDV RNA and mDV RNA. These results are akin to that observed with refolding of reduced-denatured lysozyme in presence of GroEL:GroES (in absence of ATP) where the reactivation of the protein is fully suppressed due to formation of a complex between GroEL-GroES and lysozyme folding intermediate [31]. Hence the inhibition of reactivation as observed here might also indicate the formation of a stable complex between the refolding protein and the ribosome or domain V RNA under redox buffer conditions. Indeed, digestion of RNA by RNase treatment of bDV RNA-lysozyme complex leads to lysozyme reactivation yield comparable to that of self folding (Fig. 2A). When the ribosome was treated with ribonuclease and its ability to bind and inhibit reactivation of lysozyme was assessed, no reactivation of the protein was observed probably because of limited access of the ribosomal RNA to the RNase enzyme. The ribosome was treated with Proteinase K, extracted using phenol, precipitated with ethanol and treated with RNase. The reactivation yield of the lysozyme in presence of such treated ribosome ∼47% compared to ∼82% obtained in absence of any chaperones as shown in Fig. 2A. Together these experiments indicate that stable interaction between refolding lysozyme and the domain V RNA might be responsible for lack of reactivation observed.

#### Turbidity measurements

Turbidity measurements performed at 450 nm (Fig. 2B) shows that the protein undergoes rapid aggregation during its refolding from reduced- denatured state under the non redox conditions. The fact that aggregation of lysozyme proceeds even at high concentrations of DTT (6 mM) indicate (data not shown), as reported earlier, that the early phase of aggregation proceeds due to hydrophobic interaction between protein molecules and not due to intermolecular disulfide bond formation [32]. The aggregation process is significantly suppressed in presence of the ribosome, bDV RNA or mDV RNA (Fig. 2B **)**. Evidence of complex formation between refolding lysozyme and domainV RNA or 70S ribosome under these conditions are shown below.

#### Electron microscopy

Suppression of lysozyme aggregation by bDV RNA was confirmed by electron micrograph images of reduced-denatured lysozyme under refolding condition (materials and method) in absence and in presence of bDV RNA (Fig. 2C). Lysozyme (2 µM) was incubated in absence or presence of equimolar concentration of bDV RNA under refolding conditions as stated above. The TEM pictures show that formation of large amorphous lysozyme aggregates when refolded in absence of a chaperone (Fig. 2C.i). The aggregates are absent upon incubation with bDV RNA in agreement with the data obtained by turbidity measurements (Fig. 2C.ii).

#### Effect of ribosome associated chaperones on refolding of lysozyme

The ability of the ribosome associated chaperones like the Trigger factor and DnaK chaperones to affect reactivation or suppress aggregation during refolding lysozyme was assessed in absence of any added co-chaperones or co-factors. The chaperone: protein ratio was 1∶1.

As shown in Figure S3D under non-redox conditions presence of 70S ribosome was more effective in suppressing aggregation during refolding of reduced-denatured lysozyme than either DnaK or Trigger factor. Under redox conditions, none of the studied factors (70S+DnaK+Trigger factor, 70S+DnaK+Trigger factor +ATP) could enable reactivation possibly due to failure in releasing the protein from the RNA-protein complex (Figure S3C).

### Interaction of BCAII and Lysozyme with Ribosome and its Domain V RNA

Earlier studies on bDV RNA mediated refolding of unfolded BCAII had shown that the RNA1 sub-domain of bDV RNA interacts with the refolding protein and the RNA2 subdomain is responsible for the releasing the bound protein in a folding competent state within 180 seconds from the initiation of interaction. To follow the time course of interaction between the chaperone and protein (bDV RNA, mDV RNA or ribosome with partially folded BCAII or lysozyme intermediate), the following experiments were performed.

#### Filter binding studies

To study the effect of binding and release of the bDV RNA associated protein on refolding and aggregation of BCAII-m, mutations U2585C (in the RNA1 region) and delG2252 (in the RNA2 region) were introduced in DNA corresponding to domain V RNA (materials and methods section).

**Figure 3:**
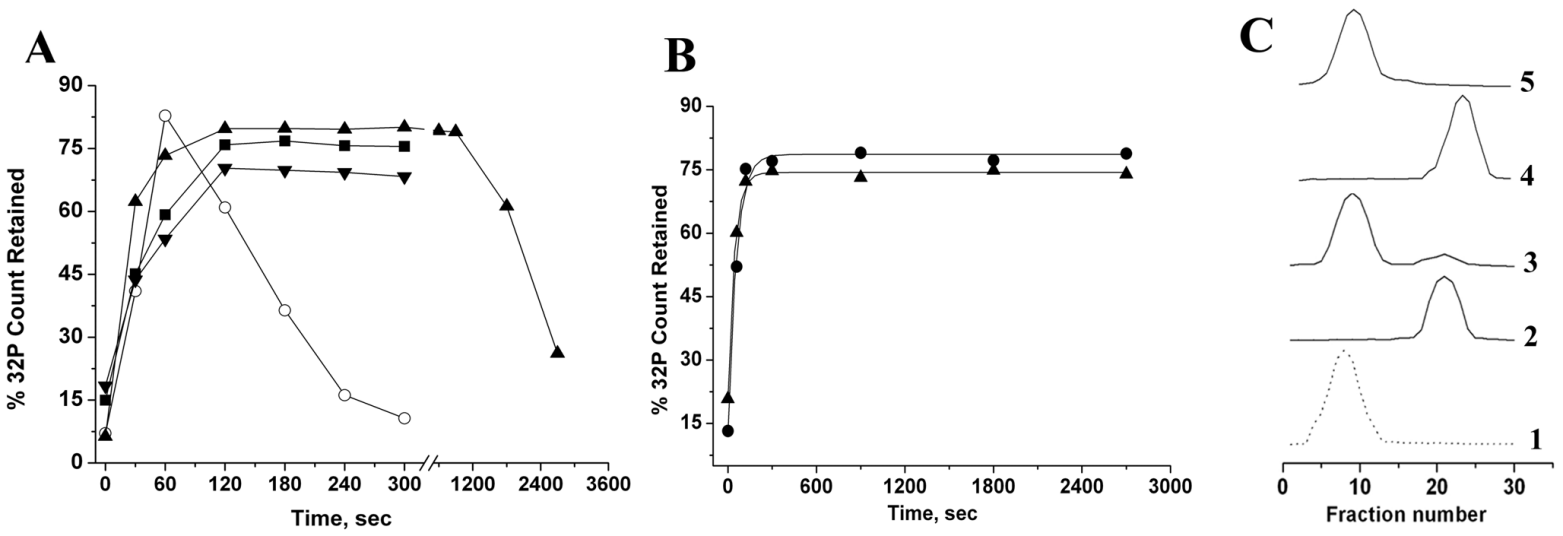
Interaction of BCAII and lysozyme with ribosome and its domain V RNA. *Filter binding studies.* Refolding of BCAII-m or reduced-denatured lysozyme was initiated in presence of radiolabeled various domain V RNA, was UV- crosslinked and filtered through nitrocellulose membrane (material and method). A) Time course of interactions of BCAII-m with radiolabeled bDV RNA (-○-), mDV RNA (-▴-), bDV RNA mutants U2585C (-▾-) and delG2252 (-▪-) are shown here. Experiments were repeated thrice and their average values were taken for final data plotting. B) Time course of interactions of reduced-denatured lysozyme and radiolabeled bDV RNA (-•-) and mDV RNA (-▴-) are shown. *Size exclusion chromatography.* Refolding of FITC labeled BCAII-m or reduced-denatured lysozyme was initiated in presence of 70S ribosome, was UV-crosslinked at 30 second of refolding, and the mix was loaded on Sephacryl S-300 column. The elution of the protein and the ribosome was monitored by fluorescence at 518 nm and absorbance at 260 nm. C) Detection of 70S- BCAII complex. The elution profiles of BCAII-m in presence of ribosome at 30 seconds of refolding (3), reduced denatured lysozyme in presence of ribosome at 30 seconds of refolding (5) are shown. The elution profiles of ribosome (1), native BCAII (2) and native lysozyme (4) are also shown for comparison.

**Figure 4:**
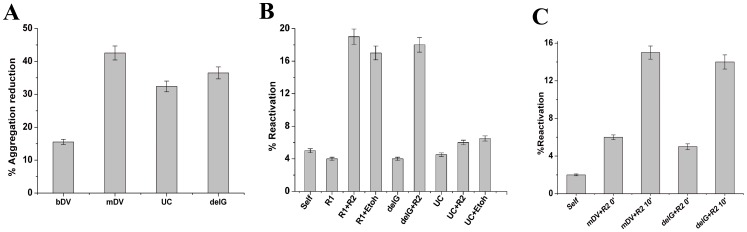
Effects of bDV RNA mutants on refolding of BCAII. A) Bar diagram shows percent aggregation reduction during BCA II-m refolding by bDV RNA, mDV RNA and bDV RNA mutants U2585C (UC), delG2252 (delG). The turbidities in each case were measured at 320 nm, 12 minutes after return to refolding conditions and turbidity in absence of chaperone was assumed as 100%. B) Comparison of reactivation yields of BCAII-m (0.3 µM) after 30 min of refolding in absence of the chaperone (Self) and in presence of bDV RNA1(R1), bDV RNA1+RNA2 (R1+R2), RNA1+3% Ethanol (R1+EtOH), del G2252 bDV RNA (delG), del G2252+ RNA2 (delG+R2), U2585C (UC), U2585C bDV RNA+RNA2 (UC+R2) and U2585C bDV RNA+3% Ethanol (UC+EtOH). C) Comparison of the reactivation yields of BCAII-m (0.9 µM) after 30 min of refolding in absence of the chaperone (Self), in presence of del G2252 bDV RNA and mDV RNA. BCAII-m reactivation upon addition of RNA 2 portion of bDV at zero minute (mDV+R2 0′and delG+R2 0′) and after ten minutes of initiation of refolding (mDV+R2 10′ and delG+R2 10′) are also shown.

The time course of interaction of BCAII-m or reduced-denatured lysozyme to bDV RNA mutants and mDV RNA were monitored by filter binding studies. Briefly, BCAII-m or reduced-denatured lysozyme was incubated with ^32^P labeled domain V RNA under refolding conditions. Aliquots of refolding mix were withdrawn at different time intervals after initiation of folding, crosslinked and passed through nitrocellulose filter. The radioactivity retained on the filter represents the RNA-protein complex present at each time point. Fig. 3A shows that binding of the BCAII is comparable with the mutants and wild type bDV RNA and is completed within about the 60 seconds after initiation of interaction. However, while bDV RNA releases the refolding protein within 300 seconds after initiation of refolding, neither of its mutants U2585C or delG2252 does. The addition of wild type RNA2 could not induce release of BCAII from the U2585C mutant (Figure S4). The effect of the delayed release of the protein from bDV RNA mutants and mDV RNA on reactivation and aggregation of BCAII-m is shown below (Fig. 4). Release of protein bound to mDV RNA was completed only after ∼45 min after initiation of refolding.

Filter binding studies with reduced-denatured lysozyme showed that neither bDV RNA nor mDV RNA could release the bound protein even after 3000 seconds after initiation of refolding under non-redox conditions (Fig. 3B). The studies shown above also demonstrate that a similar inability to release the RNA bound protein was responsible for the lack of reactivation observed in presence of the chaperones under redox refolding conditions (Fig. 2A).

#### Size-exclusion chromatograph

We wanted to study the time course of interaction of the refolding proteins with the ribosome. We incubated FITC labeled BCAII-m or reduced-denatured lysozyme with 70S ribosome in the respective refolding buffer (Materials and Method). Aliquots of the refolding mixture were withdrawn 30 seconds after initiation of refolding in presence of the ribosome, UV- crosslinked and analyzed by gel filtration. The elution of the ribosome and proteins were followed by absorbance at 260 nm and fluorescence at 518 nm respectively. The elution profile of the ribosome and the native BCAII and lysozyme were also separately determined. As shown in Fig. 3C after 30 seconds of refolding, almost all of the FITC-labeled protein eluted in the excluded volume of the column along with 70S ribosome, indicating that BCAII initially binds with a high affinity to the ribosome. Similarly, during refolding of reduced-denatured lysozyme under non-redox conditions, after 30 seconds of refolding, almost all of the FITC-labeled protein eluted in the excluded volume of the column along with the ribosome, indicating initial complete binding of refolding lysozyme to the ribosome (Fig. 3C). Similar results were obtained from studies that were performed at higher final DTT concentration (6 mM) (data not shown). Thus even under conditions mimicking the reducing environment of the bacterial cytosol, the protein was not released and remained stably associated with the ribosome.

Taken together, these studies show that the early refolding species formed during BCAII-m and lysozyme refolding interact with the ribosome and the bDV RNA. The efficient and complete binding of these BCAII intermediates to the ribosome is achieved within the first 30 seconds of return to refolding conditions compared to ∼60 seconds required with the bDV RNA. This might explain why the ribosome was more effective in suppressing aggregation and therefore increasing reactivation and highlights the importance of the time of binding to the chaperones in determining its chaperoning ability.

### Effects of bDV RNA Mutants on Refolding of BCAII

The relative extents of aggregation suppression of BCAII by the bDV RNA mutants were determined. The bar diagram (Fig. 4A) shows that the delayed release mutants of bDV RNA are more effective in aggregation suppression. The turbidities in each case were measured at 320 nm, 12 minutes after return to refolding conditions and turbidity in absence of chaperone was assumed as 100%.

The effect of bDV RNA mutant on BCAII-m reactivation studies are shown in Fig. 4B. When BCAII-m (0.3 µM) was refolded in presence of the delG2252 bDV RNA and wild type RNA2, release and reactivation of the protein was observed. No reactivation was observed either with wild type RNA2 or ethanol (3%) induced release of the protein from mutant U2585C of bDV RNA. As this nucleotide position (U2585) coincides with one of the five crucial interaction sites between RNA1 and the refolding protein, the mutant possibly fails to release the protein in a folding competent state. Thus, although, the delayed release mutants of bDV RNA were more effective in suppressing BCAII aggregation than their wild type counterpart (shown above), release of the protein from these mutants did not necessarily lead to improved reactivation. This implies that aggregation suppression and increase in reactivation during ribosome mediated refolding might represent two distinct aspects of the chaperone function.

When early release of BCAII-m (0.9 µM) from either delG2252 bDV RNA or mDV RNA was induced with wild type RNA2, marginal reactivation yield was observed. A delay of 10 minutes in addition of wild type RNA2 to BCAII-m refolding mix (Fig. 4C) leads to increase in reactivation yield. This again establishes that the time of release of the protein from the chaperone is an important criterion in determining its reactivation yield.

## Discussion

The present study is the first in which the chaperoning activities of ribosome and its domain V RNA on partially folded intermediates of proteins have been characterized and it has been demonstrated that the chaperones can suppress aggregation of the proteins that competes with refolding process. Earlier studies have shown that the ribosome can enhance solubility of aggregation prone proteins that are coupled to its surface. The intrinsic charge of the ribosomal RNA or the steric effect of association with the ribosome has been proposed to be responsible for this property [33]. Other studies have implied that the surface hydrophobicity of the ribosome may also contribute to its chaperoning ability [34]. The data presented here however indicate involvement of specific rRNA mediated mechanisms of aggregation suppression. The extent of aggregation suppression depends upon the delay time in release of the chaperone bound protein. The inability of the chaperone to release the bound intermediate either due to mutation (BCAII-m and bDV RNA mutants Fig. 4) or due to the intrinsic nature of chaperone-protein complex (lysozyme and bDV RNA/ribosome Figs. 2 and 3) prevents reactivation of the protein even under appropriate refolding condition. The partial reactivation achieved in ribosome assisted refolding of BCAII-m at high protein concentration might also indicate incomplete release of ribosome bound protein. This study therefore implies the presence of additional cellular factors that would enable release of the chaperone bound protein thus ensuring sustenance of the translational ability of the ribosome. The identification of these cellular factors requires further investigation. Whether the ability of the ribosome to bind partially unfolded proteins is relevant under stress conditions needs to be further investigated.

Recent experimental evidences suggest that the discontinuities in the rates of translation which are determined by the presence of rare codons in the mRNA might have significant effect on cellular protein folding [35]. Although the nascent polypeptide tunnel through which the protein emerges from the ribosome might limit the conformations available to the nascent polypeptide chain thereby trapping the chain in an “extended” conformation until completion of polypeptide synthesis, there is also ample experimental evidence in support of cotranslational protein folding [35]–[37]. Based on these facts and that the early events in the protein folding process occur in a timescale much faster than protein biosynthesis, it had been argued that the nascent polypeptide chain, upon emerging from the ribosome might be in an “extended” conformation or partially folded state similar to the “molten globule” state that has been observed in vitro [38]. Further, the cell might also need to maintain this state for self assembly, transmembrane transport and other processes that need protein molecules in their semi-flexible rather than in their rigid states. Previous studies have proposed that the ribosome acts as a ‘foldase’ chaperone that, via its specific RNA-protein interaction sites, provides information for the correct folding of unfolded polypeptide chains [39]. Our studies demonstrate that the ribosome, an essential organelle that is ubiquitously present, in large numbers in all living cells, has the ability to bind to partially folded but not to their completely folded state of proteins. In the above perspective, this ability might contribute towards either preserving the molten globule state of nascent polypeptide chains or preventing unproductive interactions (aggregation) between them.

## Supporting Information

Figure S1 **Structure of 23S ribosomal RNA and domain V RNA.** A) The 23S rRNA of *E. coli* large ribosomal subunit (PDB: 2I2V) has been displayed (orange). The domain V rRNA is highlighted in ribbon (grey). B) Secondary structures of domainV of large ribosomal subunit RNA of *E.coli* (bDV RNA) with RNA1 and RNA2 regions marked. The black square in RNA1 and RNA2 represents the nucleotide U2585 and G2252 respectively. (C) Secondary structures of domainV of large ribosomal subunit RNA of Bovine Mitochondria (mDV RNA).
(TIF)

Figure S2 **Delay in addition of bDV RNA reduced BCAII reactivation yield.** Refolding of BCA II-m was initiated in refolding buffer lacking domain V RNA. The chaperone was then added at the indicate times. After 30 min the samples were assayed for BCAII activity. The reactivation yield was determined with reference to equal concentration of native BCAII. The effect of time interval between initiation of refolding and addition of Domain V on reactivation yield is indicated.
(TIF)

Figure S3 **Effect of ribosome associated chaperones on refolding of BCAII and lysozyme.** A) Comparison of the reactivation yield of BCAII-m (0.9 µM) after 30 minutes of refolding in absence of chaperone (1) and in presence of 70S ribosome (2), 70S+DnaK+Trigger factor (3). B) Time course of change in turbidity at 450 nm of BCAII-m (0.9 µM) upon dilution of denaturant and in absence of chaperone (-▪-) or in presence 70S ribosome (-•-), DnaK (-▾-), and Trigger factor (-▴-) are shown. C) Comparison of the reactivation yield of reduced- denatured lysozyme (2 µM) after 16 hrs of refolding (redox buffer) in absence of chaperone (1) and in presence of 70S ribosome (2), 70S ribosome+DnaK+Trigger factor (3), 70S ribosome+DnaK+Trigger factor+ATP (4). D) Time course of change in turbidity at 450 nm of reduced-denatured lysozyme upon dilution of denaturant (non-redox buffer) in absence of chaperone (-▪-), in presence of DnaK (-▾-), Trigger factor (-▴-) and 70S ribosome (-•-) are shown.
(TIF)

Figure S4 **Binding and release of BCAII-m in the presence of wild type and mutant RNA.** The time course of binding of BCAII-m with wild type bacterial RNA1 (-▴-) and bDV RNA1 mutant U2585C (-▪-) and wild type bacterial RNA2 mediated release of the protein from wild type RNA1 (.Δ.), bDV RNA1 mutant U2585C (.□.) are shown here. The binding and release experiments were repeated thrice and their average values were taken for final data plotting.
(TIF)
