# Supplementary figures and images for: The Ribosome Can Prevent Aggregation of Partially Folded Protein Intermediates: Studies Using the Escherichia coli Ribosome
Source: PLoS One. 2014 May 7;9(5):e96425. doi: 10.1371/journal.pone.0096425 (PMC4013144; doi:10.1371/journal.pone.0096425)

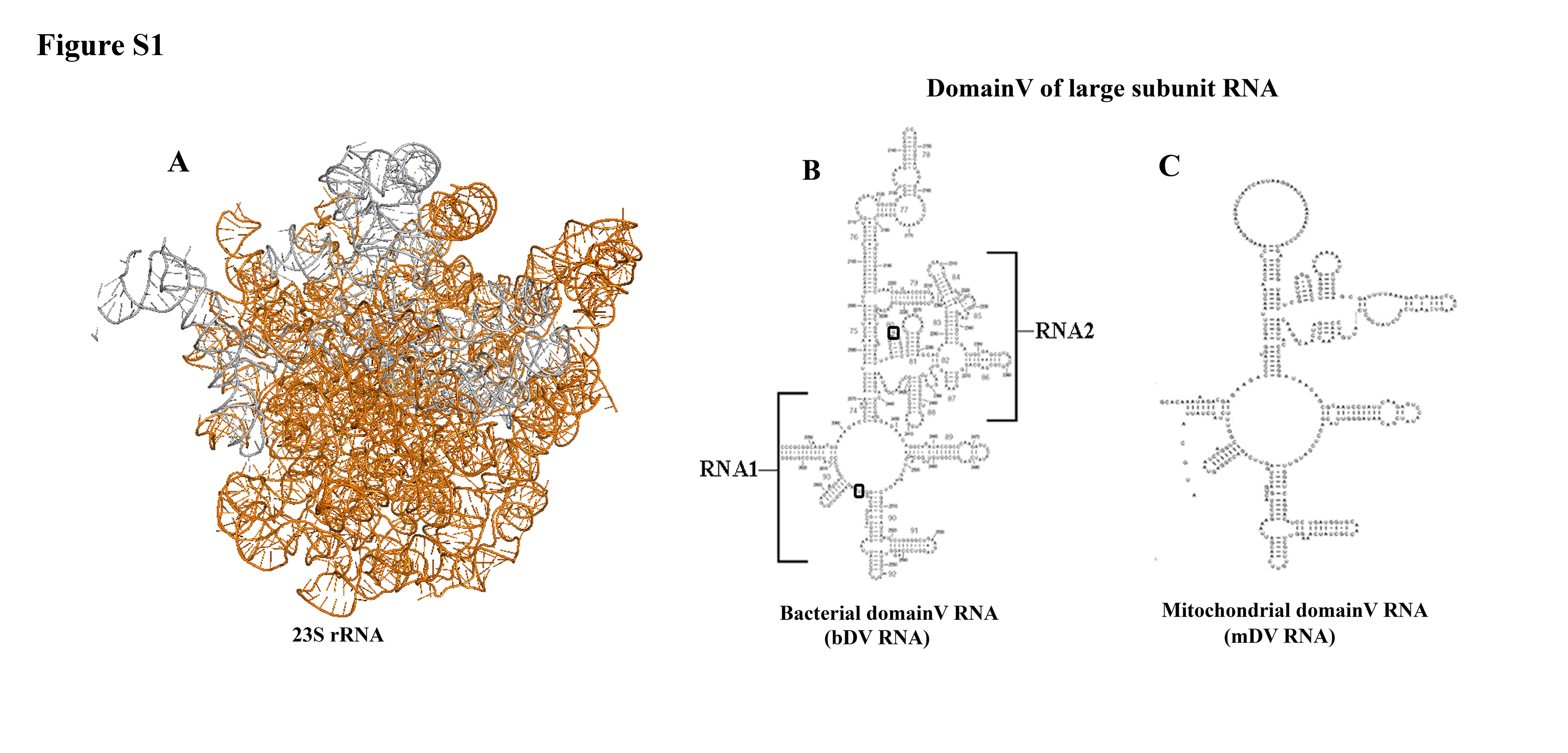

Supplement: Figure S1 — Structure of 23S ribosomal RNA and domain V RNA. A) The 23S rRNA of E. coli large ribosomal subunit (PDB: 2I2V) has been displayed (orange). The domain V rRNA is highlighted in ribbon (grey). B) Secondary structures of domainV of large ribosomal subunit RNA of E.coli (bDV RNA) with RNA1 and RNA2 regions marked. The black square in RNA1 and RNA2 represents the nucleotide U2585 and G2252 respectively. (C) Secondary structures of domainV of large ribosomal subunit RNA of Bovine Mitochondria (mDV RNA). (TIF) [file pone.0096425.s001.tif]

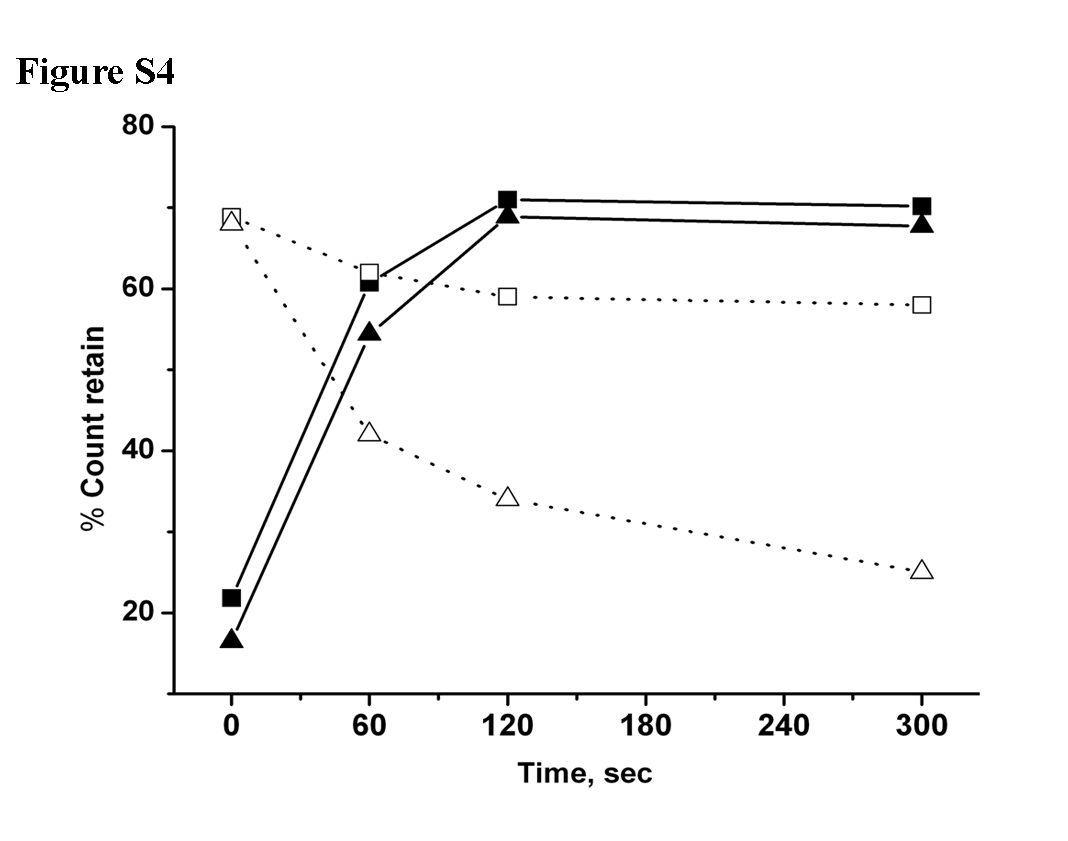

Supplement: Figure S4 — Binding and release of BCAII-m in the presence of wild type and mutant RNA. The time course of binding of BCAII-m with wild type bacterial RNA1 (-▴-) and bDV RNA1 mutant U2585C (-▪-) and wild type bacterial RNA2 mediated release of the protein from wild type RNA1 (.Δ.), bDV RNA1 mutant U2585C (.□.) are shown here. The binding and release experiments were repeated thrice and their average values were taken for final data plotting. (TIF) [file pone.0096425.s004.tif]
